# Enhancing the Ultrasonic Welding of Wood Using 3D Printed Lignin Energy Directors

**DOI:** 10.1002/advs.202507055

**Published:** 2025-09-15

**Authors:** Muhamad Amani, Kathrin Weiland, Mark Ablonczy, Natalia Sofia Guevara‐Sotelo, Ioannis Zygouris, Johan van Stuyvesant Meijen, Kunal Masania

**Affiliations:** ^1^ Shaping Matter Lab Faculty of Aerospace Engineering Delft University of Technology Kluyverweg 1 Delft 2629 HS Netherlands; ^2^ SAM XL TU Delft Rotterdamseweg 382C Delft 2629 HG Netherlands

**Keywords:** 3D printing, biobased composites, lignin, sustainable structures, ultrasonic welding

## Abstract

Ultrasonic wood welding is an ecofriendly method for rapidly joining wooden components in less than 2 s. However, this dynamic process results in low mechanical performance and poor durability under wet conditions. Inspired by natural wood's robust interlocking cellular structure, which leverages lignin fusion to enhance structural integrity, lignin fusion at wood interfaces is optimized, significantly improving lap shear strength and wet durability. These results demonstrate that enhanced lignin fusion at interfaces is crucial for obtaining strong wood joints by positioning lignin as a sustainable energy concentrator, promoting greener manufacturing of sustainable structures into complex shapes. The joints exhibit lap shear strengths and wet durability comparable to those achieved with water‐based wood and epoxy adhesives, while also demonstrating conductivity which could be leveraged for multifunctional features such as strain sensing. The approach can be extended to other manufacturing methods, such as hot‐pressing and continuous robotic manufacturing, emphasizing its potential for scalability and broad industrial adoption.

## Graphical abstract

Sustainable manufacturing for lightweight structures using ecofriendly materials will be key to reducing material consumption and lowering carbon footprints. Here, an approach is presented to weld wood using ultrasonic vibrations with material at the joint interface to direct energy. Energy directors are produced from fully biobased constituents that can be augmented with multifunctional features such as sensing to result in smart sustainable structural joints.

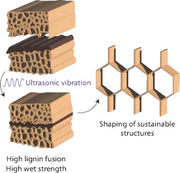

## 1 Introduction

To reduce society's environmental impact, sustainable manufacturing for lightweight structures using eco‐friendly materials and energy‐efficient processes will be key. By reducing material consumption and optimizing production techniques, we can lower carbon footprints, minimize waste, and improve energy efficiency. As global environmental concerns grow, adopting sustainable manufacturing approaches using biobased materials such as additive joining technologies could be promising.

Research has explored various adhesive joining strategies involving synthetic and bio‐based adhesives that provide strong, water‐resistant bonds, but they rely on petrochemicals and can release formaldehyde or other harmful volatiles.^[^ ^1^, ^2^ ^]^ Recent efforts have focused on starch‐based adhesives, which utilize abundant, renewable polysaccharides. These adhesives have shown promising results as eco‐friendly wood binders with enhanced water resistance by chemical and enzymatic modifications.^[^ ^3^, ^4^ ^]^ Without modification, starch adhesives are water‐sensitive and often require cross‐linking or blending with synthetic polymers to achieve better performance. A broader spectrum of formaldehyde‐ and isocyanate‐free, bio‐based wood adhesives has emerged. For example, dialdehyde cellulose with oxidation forms highly crosslinked networks with lignocellulosic substrates, yielding bond strengths comparable to commercial phenol–formaldehyde resins while remaining fully biodegradable.^[^ ^5^ ^]^ Similarly, tannin‐based adhesives achieve rapid curing and excellent water resistance.^[^ ^6^ ^]^ Recent reviews of bio‐based wood adhesives survey advances in soy‐, lignin‐, protein‐, and polysaccharide‐derived binders, highlight multiple approaches where green formulations now rival or even outperform petrochemical counterparts.^[^ ^7^, ^8^ ^]^ Another approach involves using lignin as a basis for the adhesive.

Lignin is the aromatic biopolymer that naturally binds wood fibers together and contributes to the wood's intrinsic hydrophobicity. Lignin has been investigated as a replacement for formaldehyde and epoxy resin adhesives. In recent studies, a partial substitution of phenolic resins with lignin showed potential in reducing synthetic adhesives.^[1,^ ^9^ ^]^ Using lignin as a stand‐alone wood adhesive has proven difficult because extracted lignin often lacks sufficient reactivity or polymer length, which requires chemical modification or blending with other polymers to improve strength and durability.^[^ ^9^ ^]^

Beyond these adhesives, bio‐inspired methods such as bio‐welding have emerged. Fungal mycelium interfaces represent a novel, inherently bio‐based solution. In this strategy, a wood‐degrading fungus is grown at the interface of two wood pieces. Mycelium acts as a natural binder. A thin mycelium film can bind wood veneers with lap shear strength comparable to commercial wood glue.^[^ ^2^, ^10^, ^11^, ^12^, ^13^ ^]^ However, bio‐welding with mycelium requires time for biological growth and careful control of the local environmental and nutrient conditions.

Mechanical welding is an interesting green joining technology without the need for adhesives. It is a solid‐state joining process that uses high‐frequency vibrations to create frictional heat at the interface of thermoplastic materials, causing them to soften and bond without requiring additional adhesives or fasteners.^[^ ^14^, ^15^, ^16^ ^]^ The concept of vibration‐induced joining was explored using high‐frequency vibration to generate heat and melt wood's own lignin at the interface.^[^ ^17^, ^18^ ^]^ This technique offers a fast, eco‐friendly alternative to conventional glueing, capable of bonding wood with no formaldehyde or petrochemical resins.^[^ ^17^ ^]^ Earlier work on vibrational wood welding includes linear and rotational welding, where the concept was demonstrated, but the robustness of the joints needed further improvement.^[^ ^18^ ^]^ More recent studies have focused on developing this technology in dry conditions by changing the welding parameters: force, vibration frequency, and amplitude.^[^ ^19^ ^]^ Although vibrational wood welding results in high densification at the interface and a well‐defined joint,^[^ ^20^ ^]^ the current approaches yield weak joints with poor wet strength, which has hindered their applicability in materials and structures.^[^ ^21^ ^]^ Due to the poor interface formation and local damage caused by wood cells being pushed out of the interface during welding,^[^ ^18^ ^]^ they tend to exhibit mechanical weakness such as brittleness or lower ultimate strength, compared to optimally glued joints.^[^ ^17^ ^]^

Despite the progress above, a critical gap remains in a scalable additive wood joining method that delivers high strength and robust, wet, durable performance. Traditional adhesives can ensure water‐resistant bonds, but at the cost of added chemicals. Bio‐based glues like starch or unmodified lignin alone have not yet achieved the strength and moisture durability of synthetic resins. Fungal mycelium bonding, while effective in niche applications, is incompatible with rapid assembly processes.

**Figure 1:**
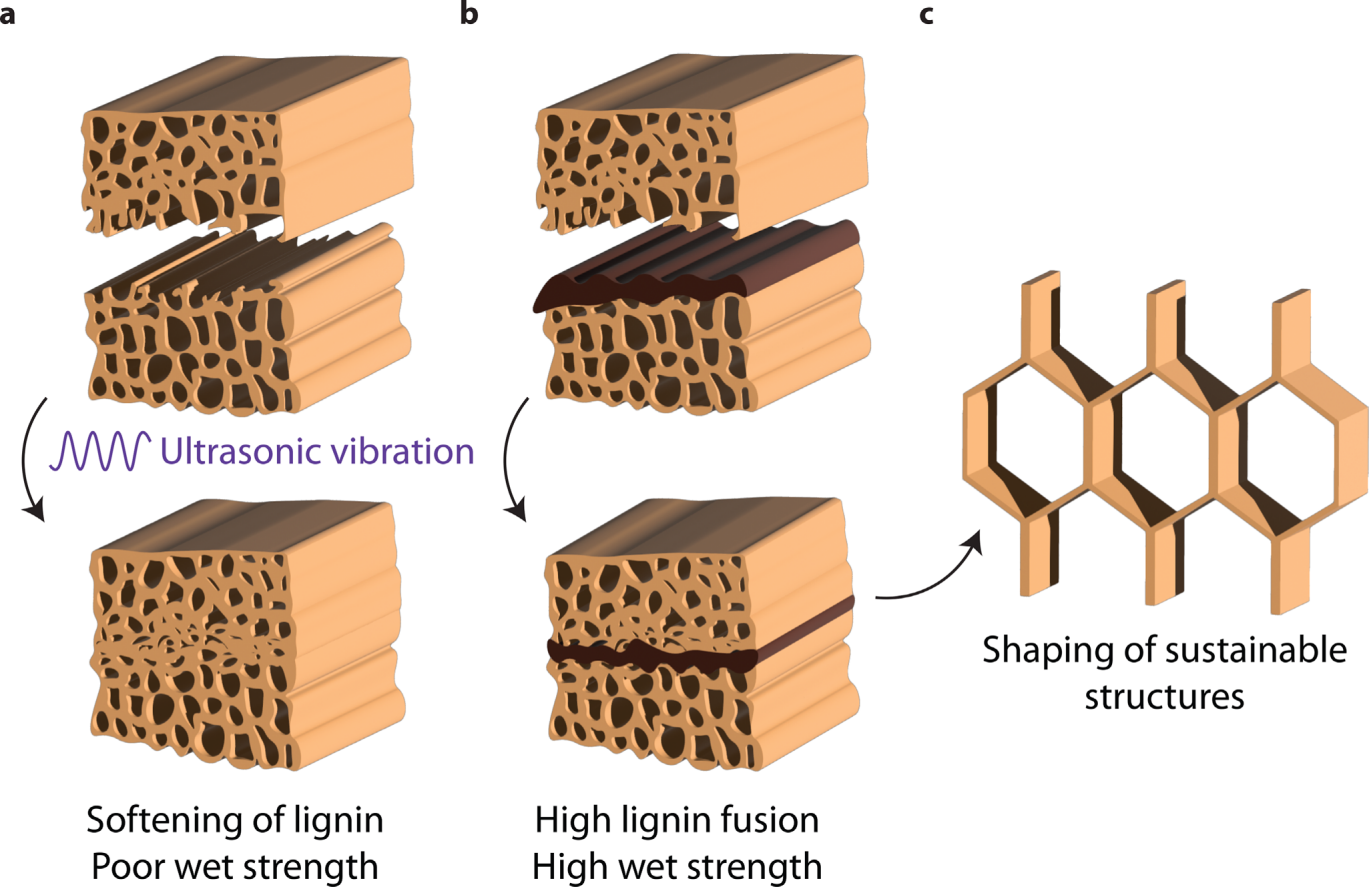
Overview of binding lignocellulosic material using ultrasonic vibration. a) Ultrasonic vibration locally softens the wood through frictional heating, resulting in fusion. b) By 3D printing a lignin‐containing energy director onto the interface, we hypothesize improved lap shear strength and wet strength. c) This method could be useful to rapidly manufacture structural complex shapes from wood materials.

We propose that, of the previously discussed approaches, ultrasonic welding could be further enhanced by introducing energy directors with compositions that mimic wood's natural composition. We hypothesize that mimicking the lignin‐based bonding and micro‐scale interlocking found in natural wood could further augment the wood's inherent bonding abilities: biopolymer adhesion and fiber entanglement. This biological analogy guides us to focus on maximizing lignin fusion and promoting an interlocking microstructure at the weld interface. A strategy we hypothesize will yield a much stronger and more water‐resistant additive joining technology, as proposed in **Figure** **1**. This approach aligns with recent advances in material‐centric additive manufacturing strategies that leverage thermal and chemical interactions to achieve enhanced functionality.^[^ ^22^ ^]^

## 2 Results

Beech wood veneer sheets (Mprofi MT, Germany) 25.4 mm × 12.7 mm with a thickness of 0.5 mm (typical lignin content ≈20 wt%) were laser‐cut to size using a Snapmaker 2.0 modular system (Snapmaker, China). The ultrasonic welding of the wood veneers was performed using a VE20 Slimline dialog 6200 machine (Herrmann Ultrasonics, Germany) equipped with a rectangular sonotrode (15 mm × 30 mm), as shown in Figure S2 and Video S1 (Supporting Information). The top and bottom veneer sheets were aligned along the fiber direction, and welding parameters, including displacement, power, force, amplitude, and frequency, were recorded for analysis. The temperature at the welding interface was measured using K‐type thermocouples (Tempco B.V., Netherlands) with a wire diameter of 0.10 mm, positioned at the overlapping center of the bottom adherend. Data were sampled at 1 kHz, with measurements repeated three times for accuracy. Additionally, a forward‐looking infrared camera (FLIR A655sc, Teledyne FLIR, USA) also recorded thermal images, which were analyzed using FLIR Research Studio software. Key thermal frames were extracted from the videos for further evaluation. Mechanical properties of the welded joints were tested using a Zwick 10 kN Universal Testing Machine (ZwickRoell, Germany) according to ASTM D1002 standards, and lap shear strength was measured for comparison with welding parameters with a bonding area of 1.27 cm × 2.54 cm. Chemical characterization of the joint interfaces was conducted using Fourier transformed infrared (FTIR) spectroscopy (Spectrum 100, PerkinElmer, USA) over a range of 400–4000 cm^−1^, with absorbance normalized via min‐max scaling using Python. Delignification of veneer samples was carried out by soaking them in a 1:1 solution of glacial acetic acid and 30% hydrogen peroxide, heating the solution to 80 °C, and stirring for 5, 10, 15, or 30 min. After treatment, the veneers were rinsed with water until the pH reached 4.5, air‐dried, and prepared for subsequent analysis.

**Figure 2:**
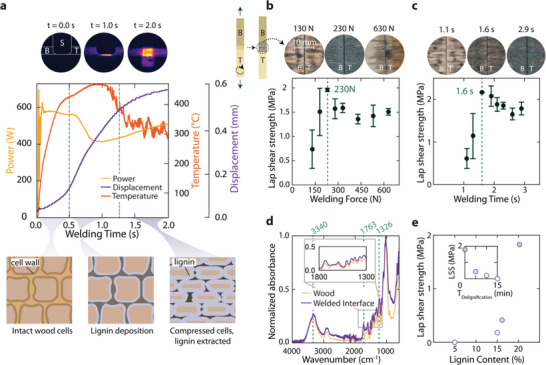
The relationship between ultrasonic welding parameters and the welded joint's lap shear strength depends on beech wood's lignin content. a) The ultrasonic welding process involves three steps: initial compression at vibration at 20 kHz to generate friction and viscoelastic heating rapidly. This high temperature increase causes lignin to soften, enables the material to flow, resulting in a displacement, and a final temperature drop indicating full cell wall compression and lignin impregnation, which can help optimize binding strength through displacement, force, and time control. b) By varying the welding force, an optimum of 230 N is obtained with respect to the final strength of the joint. Excessive force leads to over‐compression and degradation in the joint, which gives higher property variability. c) Using this welding force, we then identify a welding time of 1.6 s based on the strength of the joint. d) FTIR data of unmodified wood and welded interface. The increase in O─H stretching vibration at 3340 cm^−1^, C═O stretching vibrations in carbonyl and carboxyl groups at 1763 cm^−1^ and symmetrical bonding vibrations O─H in phenol groups at 1326 cm^−1^ suggest that more lignin is present on the interface of the weld compared to the untreated wood. e) The lignin content in wood appears to be key in governing binding during the ultrasonic welding process. Less lignin content in the wood results in lower lap shear strength.

The ultrasonic welding process is an extremely dynamic process and can be described in three key steps. Shown in **Figure** **2**a, first, an initial compression of the sonotrode to the wood joint initiates pressure and friction at the wood‐wood interface through 20 kHz vibrations of the sonotrode. The temperature at the interface between substrates increases rapidly due to the friction introduced by the vibration of the wood. The friction in the interface causes the temperature to evolve rapidly to 661 °C in 1 s. This far surpasses water phase evaporation at 100 °C, the glass‐transition temperature of lignin and other amorphous components (≈150 °C),^[^ ^23^, ^24^ ^]^ hemicellulose degradation (≈285 °C), cellulose degradation (≈350 °C), and lignin degradation, which is the most thermostable, at around 405 °C (Figure S3, Supporting Information), the first half second of the welding process.^[^ ^25^ ^]^

Under this high temperature and pressure, in the second step, the lignin softens and starts to flow between the cell walls of the interface. This results in a decrease in power and an increase in the rate of change of displacement. Since lignin does not serve its normal function of binding cellulose and hemicellulose, the mechanical integrity in the cell walls is reduced, which shows buckling and local compression of their cell walls. In the final part of the process, an unsteady decrease in the temperature inside the joint is observed. A thermal camera video shows this temperature drop on the joint's surface (Figure S4, Supporting Information). This drop indicates full compression of cell walls and full impregnation of lignin between the cell walls before consolidation. To further support this observation, porosity was quantified along the sample height using micro‐computed tomography (µCT) analysis (Figure S5, Supporting Information). A distinct reduction was observed at the interface between the two adherent layers, confirming the formation of a densified zone. This trend suggests that the process promotes compaction, leading to structural densification. These welding insights could help us control the parameters to find an optimal binding strength, which depends on the welding displacement, force and welding time.

The welding force applied by the sonotrode can be varied to result in different joint formation conditions, Figure 2b, resulting in strength changes. For enhanced shear strength, we hypothesize that compression should be optimal to sufficiently compress the interface and allow enough pressure to compress the cell walls, which locally increases the volume fraction (V_f_). We would expect this increasing V_f_ also to influence the mechanical performance of the substrate material.^[^ ^26^ ^]^ Increasing the welding force initially improves bonding strength to 2 MPa at 230 N, but eventually reduces it due to excessive pressure. These results lead to the squeezing out of fibers and a reduction in the shear strength of the joint. Further examination of the fracture surfaces reveals differences among samples subjected to varying forces. For the 130 and 230 N samples, the darker interfacial regions indicate the presence of lignin residues and a higher degradation, corresponding to stronger interfacial binding and enhanced joint strength. For the 630 N sample, the fracture interface was largely obscured due to excessive compression at the joint. This prevented a clear assessment of lignin distribution and hindered direct comparison with the interfacial characteristics of the other samples.

Similar to controlling the welding force, by increasing the welding time, Figure 2c, we allow the microstructure of the joint to evolve. We found that the lap shear strength was improved until 1.6 s in the process. Further increase in welding time resulted in excessive heat generation and degradation in the wood material. This phenomenon can also be analyzed by examining the fracture interfaces for the different lignin samples. A low lignin content at the interface, characterized by a brighter fracture surface, or excessive degradation, indicated by a darker interface, correlates with reduced lap shear strength. Notably, the fracture surface of the sample welded for 1.6 seconds exhibits an intermediate coloration, suggesting a balance between insufficient lignin presence and over‐degradation. Further chemical characterization of the fracture interface could provide deeper insights into the underlying mechanisms, leading to a FTIR spectroscopy study of the joint area.

Chemical characterization FTIR spectroscopy offers valuable insights into the fracture interface compared to unmodified wood, which is shown in Figure 2d. Based on the FTIR data, the welded wood shows an increase in O─H stretching vibration at 3340 cm^−1^, C═O stretching vibrations in carbonyl and carboxyl groups at 1763 cm^−1^ and symmetrical bonding vibrations O─H in phenol groups of lignin at 1326 cm^−1^.^[^ ^27^, ^28^, ^29^, ^30^ ^]^ The welded wood exhibits higher normalized absorbance at the mentioned peaks, indicating a greater lignin concentration at the fracture interface than unmodified wood. This change is likely driven by thermal softening and redistribution of lignin during the short but intense temperature rise in the welding process. Additionally, subtle increases in C─H and aromatic C═C stretching regions were observed, consistent with thermal reorganization of lignin and hemicellulose components.^[^ ^27^, ^28^, ^31^ ^]^ This increased lignin content likely contributes to the improved interfacial bonding. The amount of lignin in the wood is a critical parameter for the joint strength. Varying lignin contents could provide a deeper understanding and validate this finding.

Delignification of wood for varying durations allows the preparation of samples with different lignin contents. The unmodified wood was compared to wood delignified for 5, 10, and 15 minutes, as shown in Figure 2e. The lap shear strength of unmodified wood was the highest, attributed to its ability to mobilize and deposit more lignin at the interface during welding. Conversely, as lignin content decreased with increasing delignification time, a corresponding reduction in lap shear strength was observed. This clearly highlights that lignin plays a critical role in bonding wood during ultrasonic welding, suggesting that artificially increasing lignin content could further enhance lap shear strength.

**Figure 3:**
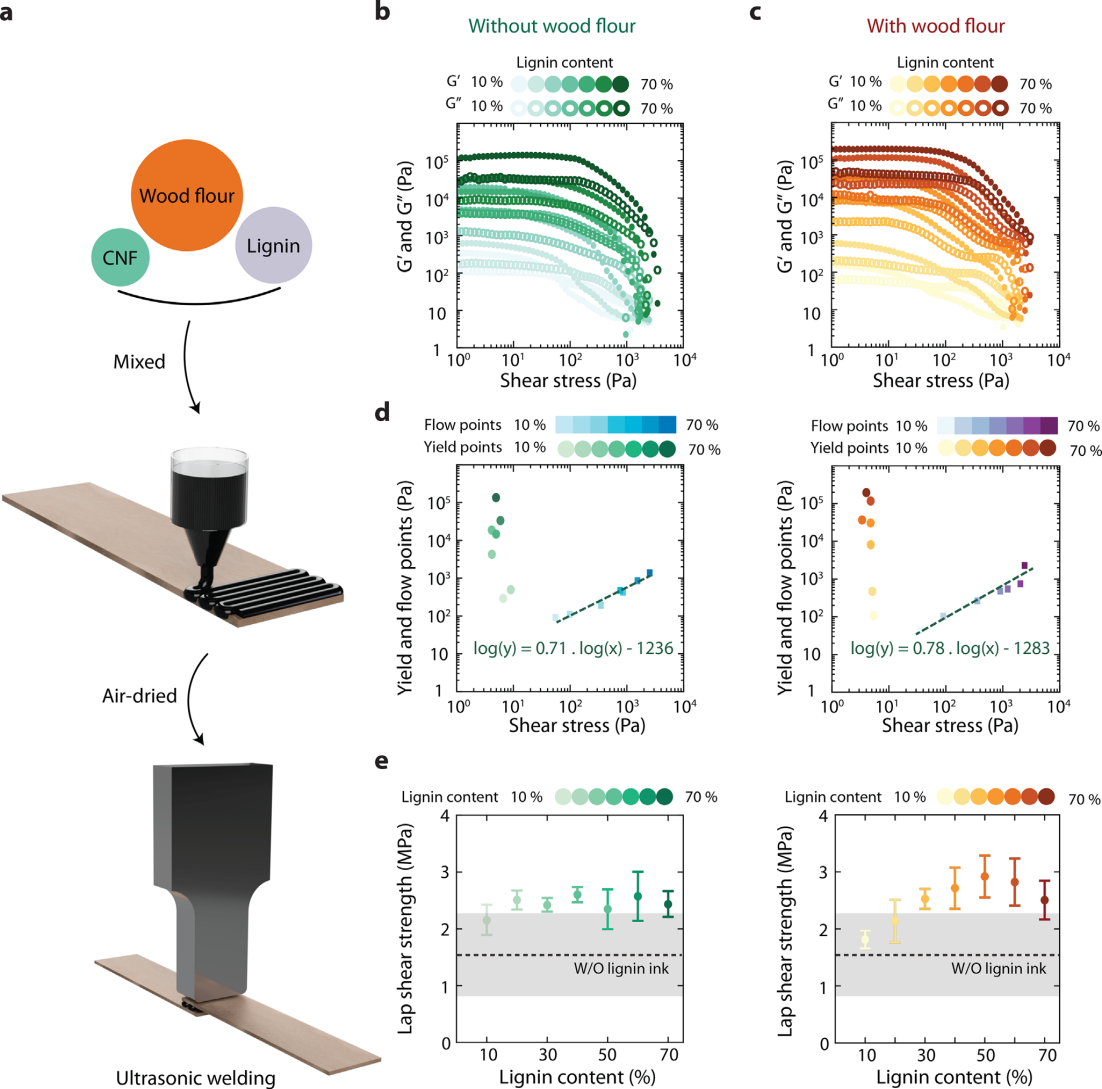
Varying the lignin content affects the rheology and the lap shear strength. a) An ink made by nanocellulose (CNFs) of varying lignin content from 10% to 70%, to direct ink writing on the wood interface. b) The impact of the lignin content in storage (*G*′) and loss modulus (*G*″). At low shear rates, *G*′ surpasses *G*″, indicating ink stability, while higher shear stresses cause yielding and energy dissipation. c) A second ink made by nanocellulose and lignin with Beechwood flour was prepared to understand the role of hydrogen bonding. d) Yield and flow points extracted for both inks show increased yield and flow points with increasing lignin content. Yield stress and flow points increase with lignin content, indicating more lignin─nanocellulose hydrogen bonding. Linear regression shows the minimal impact of wood flour on rheology. e) Lap shear strength for joints with lignin ink shows a maximum at 40% lignin while the ink with wood flour peaks at 50% lignin. These joints outperform those without energy directors, emphasizing the importance of lignin content in improving joint performance.

The 3D printing inks were formulated using Beechwood flour, cellulose nanofibers, and lignin, with varying lignin concentrations. The components were mixed for uniformity and centrifuged to remove air bubbles before printing, as shown in Video S2 (Supporting Information). Rheological properties were analyzed using a rotational rheometer. A modified 3D printer with a syringe‐based extrusion system was used to fabricate lignin‐based energy directors, which were printed onto wood veneer and air‐dried before ultrasonic welding (**Figure** **3**a and Figure S6a, Supporting Information).

For inks containing nanocellulose and lignin, Figure 3b, *G*′ exceeds *G*″ at lower shear rates, followed by a decline in both at higher shear rates. This behavior underscores the stability of the ink prior to direct ink writing (DIW) and its suitability for DIW applications. Comparing 10 wt% and 70 wt% lignin content suggests that the bonding between nanocellulose and lignin is stronger than that involving wood flour. Further rheological analysis could highlight two critical points in the mechanical behavior of the inks: the yield point and the flow point. Further investigation with an ink with wood flour could help clarify the role of nanocellulose and wood flour in forming these bonds and maintaining the structure.

For all inks with wood flour, at low shear rates, Figure 3c, *G*′ consistently exceeds *G*″. This dominance of *G*' highlights the stability of the inks and their ability to retain structural integrity prior to extrusion through the nozzle. As the shear stress increases, *G*' exhibits a decline, indicative of the yielding behavior as the ink network structure disrupts under elevated stresses. Similarly, G″ demonstrates a proportional reduction, reflecting energy dissipation mechanisms that vary with lignin composition. The transition from 10 wt% to 70 wt% lignin content reveals a pronounced increase in elastic response at higher lignin concentrations. This non‐linear rheological behavior is likely attributable to the formation of hydrogen bonds between the hydroxyl and carboxyl groups of lignin and the hydroxyl groups present in nanocellulose and wood flour, as supported by the findings of Borghei et al.^[^ ^32^ ^]^ The yield behavior and shear stress response were extracted from the data to gain further insights into the different prepared inks, as shown in Figure 3d.

The yield point (τ_y_) marks the transition from an elastic‐dominant regime, where the material resists deformation and exhibits a structured network, to a fluid regime, where the internal structure partially breaks down under stress, allowing the material to start flowing. The flow point (τ_f_) is characterized by the intersection of *G*’ and *G*″. By extracting the yield points and flow points, both plots clearly indicate that higher lignin content shows higher yield points and flow points. Linear regression analysis (Figure 3d) shows that the slope for inks without wood flour is 0.71, while for inks with wood flour, it is 0.78. This suggests that the presence of wood flour has a minimal impact on increasing the yield point of the ink. Both inks demonstrate suitability for DIW onto adherent surfaces, serving as energy directors to enhance bonding during ultrasonic welding. This could effectively concentrate ultrasonic energy at the bonding interface. The inks' rheological properties ensure precise deposition, while their composition facilitates optimal energy absorption and heat generation. This dual functionality could enhance the efficiency and reliability of ultrasonic welding, making these inks highly effective in composite assembly applications. Samples were prepared by depositing inks onto the interface of wood samples, prior to ultrasonic welding. Our hypothesis is that the ink can serve to distribute vibrational energy more effectively over the joint interface, whilst also providing more lignin in the forming interface region of the joint.

The lap shear strength of joints fabricated using lignin‐based inks without wood flour increases with lignin content, reaching a maximum at 40 wt%, as shown in Figure 3e. The maximum lap shear strength of joints produced with lignin‐based inks containing wood flour is observed at a higher lignin content of 50 wt%. Notably, both lignin‐based formulations demonstrate superior lap shear strength compared to those fabricated without inks, which highlights their importance as energy directors and interface filling and bonding adhesives. Adding to the energy director, wood flour enhances lap shear strength relative to inks without wood flour, which may be attributed to improved physical and chemical interactions at the joint interface. To investigate this, a cross‐sectional analysis of the welded joints was performed (Figure S6, Supporting Information). As reported by Borghei et al.,^[^ ^32^ ^]^ wood flour particles can align during 3D printing based on the shear of the nozzle. In the present study, a similar alignment may occur during welding as well, leading to improved interfacial bonding. Microscopic and SEM images (Figure S6, Supporting Information) also confirm the presence of ordered wood flour particles within the joint. To further clarify the chemical interactions between the inks and the interface, FTIR spectra of both ink formulations were compared to that of a joint fabricated without ink (Figure S7, Supporting Information). The FTIR spectra indicate that both lignin‐based inks exhibit higher normalized absorbance values compared to the joint without ink. However, the difference between the ink with and without wood flour is insignificant, suggesting that physical rather than chemical phenomena are the main reason for improved interface bonding. The modulated differential scanning calorimetry (mDSC) results (Figure S8, Supporting Information) show that both lignin‐based inks exhibit a glass transition temperature (*T*_g_) in the same range as lignin itself, indicating that the incorporation of wood flour does not significantly alter the polymeric relaxation behavior in the lignin. The presence of wood flour slightly shifts the transition temperatures, which may be due to physical interactions between the lignin matrix and the wood particles. The thermogravimetric analysis (TGA) results (Figure S9, Supporting Information) further reveal that lignin‐based ink without wood flour exhibits higher thermal stability in the range of 300 °C to 600 °C compared to the formulation containing wood flour by having a higher normalized weight. This suggests that the wood flour component introduces additional thermal degradation pathways, likely due to the wood flour's decomposition of cellulose and hemicellulose components. This lack of chemical interaction may be attributed to the rapid fabrication process, where the entire process is concluded in 1.6 s of welding, followed by a 4 s consolidation time. To gain further understanding of the effect of short time duration, the fabrication time was extended for a new set of samples by incorporating a hot‐pressing step.

**Figure 4:**
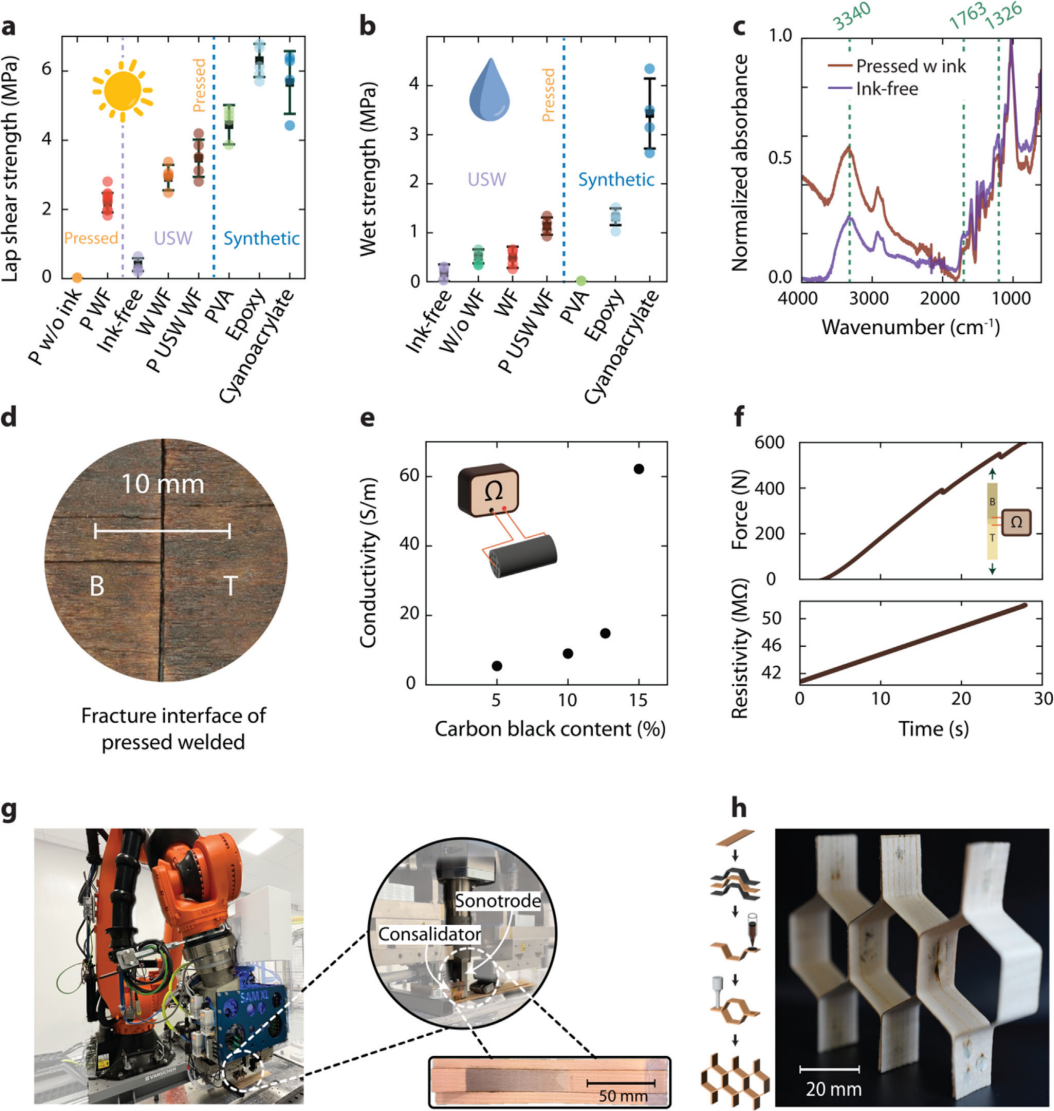
Comparison of lignin‐containing ultrasonic welding to current state of the art and possible use cases. a) The results are grouped into three categories: pressed, ultrasonic welding (USW), and synthetic adhesives. The pressed group includes samples pressed without lignin ink (P w/o ink) and with lignin ink with wood flour (P WF). The USW group comprises ink‐free welded samples (Ink‐free), welded samples containing lignin ink with wood flour (W WF), and those that were both pressed and ultrasonically welded with lignin ink with wood flour (P USW WF). The Synthetic group includes benchmark adhesives: polyvinyl acetate (PVA), epoxy, and cyanoacrylate. Pressing the welded samples further increases the lap shear strength. This indicates that extended consolidation time strengthens the bond between the wood interface and the lignin ink with wood flour, making it a promising alternative to PVA wood glue in dry conditions. b) The wet strength of pressed and welded with wood flour also shows promising results compared to only welded samples and commercial wood glue. c) FTIR spectra comparing the pressed‐welded interface and ink‐free‐welded interface shows a significant change in the chemical characterization of the fracture interface of the joint. d) Microscopic analysis of the fracture interface of pressed and welded with wood flour samples indicates a reduced thermal degradation and enhanced lignin fusion, resulting in a more cohesive and uniform bond. e) Functionalizing the ink by adding plant‐based carbon black results in an increase in conductivity, which enables the formation of a functional smart interface material capable of strain sensing. f) The strain‐sensing functionality of this smart interface material enables potential applications in diverse environments, particularly in high‐humidity conditions where conventional adhesives may degrade. g) The scalability of this fabrication method is demonstrated through robotic‐assisted continuous ultrasonic welding manufacturing, which accelerates production rates and expands the potential for industrial applications. Integrating a welding and consolidation system at the robotic arm's end effector facilitates precise, automated processing for large‐scale production. h) A proof of concept honeycomb structure fabricated using lignin‐based ink showcases the potential for further advanced structural applications with complex shapes.

To assess the effect of manufacturing time on the mechanical performance of the joint, the samples with 50 wt% wood flour, were hot‐pressed for 15 min at 180 °C and 3 MPa. This extended processing duration allows the diffusion of lignin into the wood interface as the lignin undergoes softening. Thakur et al.^[^ ^33^ ^]^ reported, that after 140 °C, lignin can flow on the wood interface and bind the other wood components and mDSC data proves the same process as well (Figure S3, Supporting Information) As shown in **Figure** **4**a, the optimied manufacturing process (ultrasonic wood welding and hot‐pressed) resulted in a lap shear strength of 3.48 ± 0.54 MPa, which is comparable to commercial water‐based wood adhesives (4.45 ± 0.57 MPa). These findings highlight that lignin‐based ink, in combination with welding and subsequent hot‐pressing, can be a sustainable adhesive alternative. To further understand the bonding performance and failure mechanisms, we examined the fracture surfaces of lap‐shear specimens joined by different adhesives. As shown in Figure S10 (Supporting Information), distinct failure modes were observed. Welded joints with lignin‐based ink and epoxy specimens predominantly exhibited stock‐break failure at the edge of the interface, likely due to stress concentrations known as the bathtub effect.^[^ ^34^ ^]^ In contrast, wood glue showed cohesive failure, attributed to its deeper penetration into the wood, which increases wood–glue adhesion. Cyanoacrylate showed mixed adhesive and stock‐break failure, likely due to insufficient wetting from rapid curing.^[^ ^35^ ^]^ These results highlight the differences in bonding behavior and underscore the robust performance of the welded interfaces under mechanical loading. Further investigation into its performance under different environmental conditions, such as high humidity or wet exposure, is appropriate to assess its broader applicability in applications.

To evaluate the wet strength of different ink formulations and manufacturing techniques relative to synthetic adhesives. All samples were submerged in water at 22 °C for 24 h, followed by immediate lap shear testing upon removal, as reported in Figure 4b. The water absorption of the samples was found to be approximately 70%. The welded samples with subsequent hot pressing exhibited a promising result of 1.13 ± 0.18 MPa, which is remarkably close to the wet joint strength of epoxy bonded samples at 1.32 ± 0.17 MPa. The water‐based PVA wood adhesive exhibited no measurable wet strength, presumably as the glue fully dissolved. To contextualize these results within standardized benchmarks, the measured dry and wet lap‐shear strengths were interpreted relative to widely accepted engineering benchmarks. Although the dry state strength falls below the commonly referenced 10 MPa threshold for high‐performance non‐structural adhesives,^[^ ^36^ ^]^ it remains within the range observed for commercial water‐based systems and demonstrates adequate bonding under ambient conditions. The wet‐state results reflect sufficient moisture resistance for interior use applications. The testing conditions we used are more severe than typical short‐term soak protocols,^[^ ^37^, ^38^ ^]^ suggesting that the adhesive formulation offers promising baseline performance in indoor environments with intermittent humidity.^[^ ^39^ ^]^ These results further validate the potential of lignin‐based adhesives as an alternative to conventional wood adhesives. An in‐depth chemical analysis of the fracture interface is necessary to elucidate the underlying mechanisms responsible for the enhanced mechanical performance of welded‐pressed samples.

An FTIR analysis was conducted to compare the chemical composition of welded and hot‐pressed joints with an ink‐free sample subjected only to ultrasonic welding. The FTIR spectra revealed a significant increase in normalized absorbance values for the welded‐hot‐pressed sample, indicating a higher degree of lignin fusion at the interface. This suggests that prolonged processing time allows greater lignin diffusion, facilitating improved interfacial bonding. Specifically, the FTIR spectra exhibited increased O─H stretching vibration at 3340 cm^−1^, along with C═O stretching vibrations in carbonyl and symmetrical bonding vibrations of O─H in phenol groups at 1326 cm^−1^. These findings emphasize the enhanced presence of lignin at the fracture interface.

Beyond physical diffusion, the improved wet lap shear strength observed in samples subjected to combined ultrasonic welding and pressing is plausibly linked to cross‐linking phenomena within the lignin matrix. Previous studies by Xiong et al.^[^ ^40^, ^41^ ^]^ and Ghahri et al.,^[^ ^42^ ^]^ demonstrate significant improvements in mechanical strength and water resistance through chemically induced cross‐linking reactions. Ghahri and Park^[^ ^43^ ^]^ reported the efficacy of thermal treatment in promoting ether bond formation and stable network structures within lignin‐based adhesives, which FTIR, nuclear magnetic resonance (NMR), X‐ray photoelectron spectroscopy (XPS), and thermal analyses confirmed. Collectively, these observations underline the critical role of cross‐linking in enhancing the mechanical properties and durability we observe in our results. In line with this, Dolan et al.^[^ ^44^ ^]^ showed that partial lignin depolymerization and the formation of cinnamyl alcohol end groups during thermal treatment can create a reactive interface promoting radical coupling. This process, alongside cellulose reorganization and the loss of hemicelluloses, contributed to improved bonding performance under hot pressing. In the context of linear friction welding, Stucki et al.^[^ ^21^ ^]^ found that unmodified kraft lignin consistently outperformed its modified derivatives. This superior performance was due to its inherently high molecular weight and condensed polymeric architecture, which promotes chain entanglement, mechanical interlocking, and a dense physical network at the bond. Improved moisture resistance was due to enhanced hydrogen bonding potential from the preserved phenolic structure.

To evaluate the improvement in mechanical properties in this study, a microscopic examination of the fracture interface revealed minimal thermal degradation and milder brown coloration, further supporting the hypothesis of improved lignin fusion. The adaptability of this ink in both welding and pressing processes suggests its potential for functionalization with additional particles, such as plant‐derived carbon black nanoparticles, to enhance its properties further.

To introduce electrical conductivity, carbon black nanopowder was incorporated into the lignin‐based ink. As shown in Figure 4e, increasing the weight fraction of carbon black resulted in a proportional increase in conductivity. At 15 wt% carbon black, a conductivity of approximately 60 S m^−1^ was achieved without compromising lignin content, which was maintained at 40 wt%. This functionalized ink enables various potential applications, including strain and damage detection. The ink was employed once again as an energy director for ultrasonic wood welding to demonstrate the functionalized ink. To enable this, copper wires connected the bottom and top adherent to a multimeter, allowing resistance measurements during lap shear testing. As shown in Figure 4f, increasing the applied force resulted in a corresponding increase in resistance, demonstrating the ink's potential as a strain sensor. These findings pave the way for scaling up the manufacturing process to explore industrial applications of sustainable structures. In addition to sensing, the integration of conductivity through 3D printing has also shown promise in emerging electrocatalytic applications and flexible energy storage, where precise control over material composition and architecture is critical.^[^ ^45^, ^46^ ^]^

The feasibility of automated large‐scale production, robotic‐assisted continuous welding was employed for wood adhesion.^[^ ^47^ ^]^ Continuous ultrasonic welding can reach up to 50 mm s^−1^, thus yielding the fastest production rates for structural joints. A 25 mm wide wood veneer was successfully welded to a 50 mm wide wood laminate over a length of 100 mm with an amplitude of 74% at a speed of 2 mm s^−1^ with a sonotrode force of 230 N and a consolidator force of 500 N, as shown in Figure 4g and Video S3 (Supporting Information). This approach highlights the potential of ultrasonic welding for various structural applications. Moreover, wood shaping with welding facilitates the fabrication of more complex geometries, such as cellular architectures.

As a demonstration of its structural versatility, a honeycomb structure was first fabricated by reshaping wood veneers, followed by lignin 3D printing of energy directors and ultrasonic welding. Omega stringer shapes were first formed by molding wet veneers and holding them to shape through a drying process of 50 °C for 12 h. Then, a structure was produced by sequentially joining veneers until a three‐cell honeycomb shape was achieved, as shown in Figure 4h. This structure had a weight of 6.6 g and could withstand approximately a compressive load of 100 kg, as shown in Video S4 (Supporting Information), thus achieving a substantial compressive strength of approximately 1.95 MPa. This is favorably comparable to engineering honeycomb core materials such as Toray 3003 aluminum honeycomb and Hexcel's PAMG‐XR1 aerospace‐grade honeycomb, which typically exhibit compressive strengths between 0.8 and 10 MPa depending on density. Notably, this performance was achieved using a simple wooden architecture, demonstrating that complex shaping and high mechanical efficiency can be realized through bio‐based materials combined with lightweight design strategies. To further explore the multifunctionality of such systems, we introduce fungal growth into the welded wood structure.

Mycelium‐based composites, which rely on fungal colonization of lignocellulosic substrates, offer a promising route toward engineered living materials.^[^ ^48^ ^]^ To test the compatibility of our welded interfaces with such systems, we investigated whether the lignin‐based welds could retain their mechanical integrity during and after mycelium growth under high‐humidity conditions. After 18 days of incubation, only the samples welded with lignin‐based ink remained intact and suitable for mechanical testing (Figure S11, Supporting Information). Both groups without lignin ink, regardless of fungal inoculation, disintegrated during handling in their wet state, confirming the critical role of the ink in maintaining wet strength. Among the lignin‐welded samples, no visible degradation or loss in interfacial strength was observed in the presence of fungal growth. Mechanical testing in Figure S11 (Supporting Information) showed no significant difference in wet strength between the fungus‐exposed and control groups. This suggests that the lignin‐based welded interfaces remain structurally stable despite prolonged exposure to high humidity and lignin‐degrading fungi, highlighting the robustness of the weld under biologically active conditions.

These findings support the potential use of this welding approach in the fabrication of living wood‐based composites, where high humidity and biological activity are typically incompatible with mechanical integrity. The ability to retain interface strength under fungal colonization opens opportunities for integrating structural and biological functionality in engineered living materials.

## 3 Conclusion

Lignin containing inks as energy directors can positively influence the adhesion of ultrasonically welded wood joints. The dry and wet strength were both improved through improved adhesion, reduced material degradation and chemical reactions in the lignin present at the material interface. We have demonstrated that the approach is scalable and can produce robust, smart adhesive‐free wood joints. We found that the lignin can concentrate vibrational energy, locally flow to improve adhesion and crosslink chemically. By clarifying the crucial role of lignin as an energy director in ultrasonic wood welding, this work opens pathways for adopting fully bio‐based joining technologies, advancing sustainable materials and manufacturing techniques. The concept of lignin fusion optimization introduced here could be generalized and adapted to other manufacturing methods, such as hot‐pressing and robotic manufacturing, offering potential scalability for broader industrial applications. Overall, this work supports the development of sustainable manufacturing processes and lowering the environmental impact of wood‐based constructions.

## Experimental Section

The overall methodology followed in this study is summarized in Figure S1 (Supporting Information). This flowchart outlines the sequential steps, starting from ultrasonic welding of wood and parameter optimization, to ink formulation, characterisation, performance benchmarking, and demonstration of application potential.

### Welding

Beech wood veneer sheets (Mprofi MT, Germany) with dimensions 25.4 mm × 12.7 mm with an approximate thickness of 0.5 mm were utilized as adherents. The sheets were laser‐cut to the specified dimensions using a Snapmaker 2.0 modular system (Snapmaker, China). The top and bottom adherents were placed along the fiber direction, shown in Figure S1 (Supporting Information). The experiments were conducted with a VE20 Slimline dialog 6200 ultrasonic welding machine from Herrmann Ultrasonics (Karlsbad, Germany), following a design of experiments approach. The welder was equipped with a rectangular sonotrode with a contact area of 15 mm × 30 mm and a gain of 1:1.7 and a booster with a gain of 1:2. The samples were welded with an amplitude of 88% with trigger and touch forces of 130 N. For all the samples, the consolidation force and time were 500N and 4 seconds, respectively. After welding, process data, including displacement, power, force, amplitude, and frequency, were exported for analysis. All welding procedures were conducted under controlled environmental conditions at ≈22 °C and ≈45% relative humidity.

### Temperature Data

The temperature at the welding interface was measured using K‐type thermocouples (GG220‐2k‐0, Tempco B.V., Bodegraven, The Netherlands) with a wire diameter of 0.10 mm and an encapsulated tip diameter of 0.70 mm. The thermocouple's measuring tip was precisely positioned at the center of the overlap on the bottom adherend to capture accurate temperature data. These thermocouples were connected to an analogue amplifier, and measurements were sampled at 1 kHz. Temperature recordings were repeated three times for reproducibility. The raw thermocouple data were synchronized with welding process data and processed using a Python‐based analysis pipeline.

The temperature development at the joint interface was monitored using an infrared (IR) camera (FLIR A655sc IR, Teledyne FLIR LLC, USA). Thermal recordings were captured and analyzed using FLIR Research Studio software (Teledyne FLIR LLC, USA). Key frames were extracted from the thermal video as screenshots for further analysis. For this particular measurement, the samples were welded without consolidation.

### mDSC

The glass transition temperature (*T*_g_) was measured using (mDSC) on an MDSC250 (TA Instruments) with standard hermetic pans. The analysis involved a temperature ramp from 50 °C to 200 °C, with a heating rate of 3 °C per minute. To enhance resolution, a temperature modulation with an amplitude of 1 °C and a period of 60 seconds was superimposed onto the primary heating ramp. All samples, including kraft lignin, wood flour, and lignin ink (with and without wood flour), were pre‐dried at 100 °C. Two heating cycles were performed, with T_g_ determined from the second cycle. The glass transition temperature was analyzed using Universal Analysis 200 software (TA Instruments) via the inflection point method based on changes in the reversing heat flow signal.

### µCT and Porosity Analysis

µCT scans were performed using a CoreTOM system (TESCAN XRE, TU Delft) in cone‐beam geometry. The sample was scanned over 360° with 1440 projections at 100 kV and 75 W, using a flat‐panel detector (XRD4343, 1920 × 1896 pixels) with an effective voxel size of 28.13 µm. Reconstruction was done via filtered back‐projection with ring artefact correction.

The reconstructed volumes were exported as binary stacks using Fiji. Directional porosity was then computed following the method and code provided by Sun et al.^[^ ^49^ ^]^

### Mechanical Testing

Following data acquisition, the welded joints were mechanically tested using a Zwick 10 kN Universal Testing Machine (ZwickRoell, Ulm, Germany) in accordance with ASTM D1002 standards.^[^ ^50^ ^]^ The lap shear strength was measured and reported alongside the welding parameters for subsequent analysis. All the tests were conducted under controlled environmental conditions at 22 °C and 45% relative humidity.

### FTIR

The chemical characterization of the joint interfaces was scanned with a Fourier transform infrared (FTIR) spectrometer (100 FTIR Spectrometer, Perkin Elmer, USA). Spectra were recorded in the range of 400–4000 cm^−1^ with a resolution of 2 cm^−1^, a data interval of 0.5 cm^−1^ and 64 accumulations per scan. The absorbance data and the corresponding wavenumbers were exported for post‐processing. Min–max normalization of the absorbance data was implemented using a Python script, as defined by the formula:

(1) $$\mathrm{Normalized}\;\mathrm{absorbance}\;=\frac{A-A_{\min}}{A_{\max}-A_{\min}}$$

where *A* represents the absorbance value, and *A*_min_ and *A*_max_ represents the minimum and maximum absorbance values, respectively.

### Delignification

The delignification process for wood veneer adherents was adapted from a protocol designed for natural fiber composites (NFCM).^[^ ^51^ ^]^ Wood veneer samples were soaked in a solution comprising glacial acetic acid and hydrogen peroxide (H_2_O_2_, 30%) mixed at a 1:1 volume ratio. The veneers were completely submerged in the solution within a glass beaker and left to infiltrate overnight. The solution was then heated to 80 °C and stirred using a magnetic stirrer for varying durations of 5, 10, 15, and 30 min, depending on the experimental condition.

After the designated delignification time, the veneers were thoroughly rinsed with fresh water until the pH of the rinsing water reached approximately 4.5. The samples were placed on a metal holder to ensure structural integrity during handling. Finally, the veneers were left to air dry before further analysis.

### Lignin Quantification

The acid‐insoluble lignin content of previously delignified wood veneers was determined following the TAPPI T 222 standard procedure for Klason lignin.^[^ ^52^ ^]^ Approximately 1 g of oven‐dry sample was placed in a 100 mL glass beaker and macerated in 15 mL of cold (10–15 °C) 72% sulfuric acid. The mixture was stirred until fully dispersed, covered with a watch glass, and kept in a 20 °C water bath for 2 hours with occasional mixing. After complete dissolution, the mixture was transferred into a 1 L Erlenmeyer flask containing 400 mL of distilled water and further diluted to a final volume of 575 mL (resulting in ∼3% sulfuric acid concentration). The suspension was boiled for 4 hours while maintaining the volume with hot water. After cooling and settling overnight, the acid‐insoluble lignin was separated by vacuum filtration through a cellulose filter, thoroughly washed with hot water, and dried at 105 °C to constant weight. The residue was then cooled in a desiccator and weighed to determine lignin content as a percentage of the original dry mass.

The lignin content (wt%) was calculated using:

(2) $$\mathrm{Lignin}\;\;\left(\%\right)=\frac{A\times 100}{W}$$

where *A* is the weight of acid‐insoluble lignin (g), and *W* is the oven‐dry weight of the sample (g).

### TGA

Thermal stability analyses of the lignin, beech wood, and lignin‐based ink with and without wood flour samples were performed using a TGA 40000 thermogravimetric analyzer (PerkinElmer). Powdered samples weighing approximately 10–20 mg were placed in ceramic crucibles and heated from ambient temperature to 600 °C at a heating rate of 10 °C min^−1^ under nitrogen. The resulting thermograms were normalized based on the initial sample mass.

### Preparation of the Inks

The inks used in this study for 3D printing were composed of three primary components: wood flour, cellulose nanofibers (CNFs), and lignin. The wood flour was sourced from Weber (Netherlands). Before use, the wood flour was sieved using a 250 µm mesh to ensure uniform particle size and remove larger particulates. The CNFs were supplied by Nanografi, act as a reinforcing agent in the ink, enhancing its mechanical stability and network integrity during extrusion and after deposition. Lignin was obtained from Sigma‐Aldrich (product number 370959), a kraft lignin with high purity. For the inks containing wood flour, the formulations consisted of deionized water (40 g L^−1^), cellulose nanofibers (CNFs, 1 g L^−1^), lignin (4 g L^−1^, 8 g L^−1^, 12 g L^−1^, 16 g L^−1^, 20 g L^−1^, 24 g L^−1^, and 28 g L^−1^, corresponding to 10 wt%, 20 wt%, 30 wt%, 40 wt%, 50 wt%, 60 wt%, and 70 wt%), and wood flour (5 g L^−1^, fixed across all formulations). For the inks without wood flour, the formulations consisted of deionized water (40 g L^−1^), CNFs (1 g L^−1^), and the same lignin concentrations (4  to 28 g L^−1^). The components were mixed in a planetary mixer (SpeedMixer DAC 150.1 FVZ) at 3500 rpm for 1.5 min in 30 s intervals with a 15 s break in between to prevent excessive heating of the components during high‐speed mixing. Before printing, the cartridge was centrifugated (Z306, Hermle) at 2000 rpm for 1 min to remove any entrapped air.

### Rheology of the Inks

The rheological properties were determined using a rotational rheometer (HAAKE MARS Rheometer, Thermo Scientific). Approx. 1 g of sample was positioned between serrated plates with a diameter of 20 mm serrated plate geometry at a distance of 1.2 mm. Shear storage and loss moduli were determined as a function of shear strain via dynamic amplitude sweeps at a fixed frequency of 1 Hz with a stress sweep. The yield point was defined as the end of the linear viscoelastic region (LVE), while the flow point represented the cross‐over point where the storage and the loss modulus were equal. The viscosity (η) was determined using an increasing shear rate of 0.1 to 100 s^−1^.

### Direct Ink Writing

Lignin‐based energy directors were fabricated using a modified Ultimaker 2+ desktop 3D printer. The printer was adapted to accommodate a custom extrusion system, replacing the original print head with a mechanically driven syringe pump capable of holding 25 mL syringes. This modification followed the approach described by Gantenbein et al.^[^ ^12^ ^]^ The design and generation of the print paths for the energy directors were performed using Grasshopper (Rhinoceros, Robert McNeel & Associates), allowing for control over grid configurations. The custom scripting enabled the 3D printing of energy director structures with a nozzle diameter of 0.84 mm and a line gap of 1.65 mm. Printing was conducted at a consistent head velocity of 20 mm s^−1^, depositing the material directly onto the bottom adherent wood veneer. After printing, the samples were air‐dried in an oven for 15 min at 70 °C to ensure structural stability and readiness for subsequent ultrasonic welding experiments.

### SEM

Scanning electron microscopy (SEM) was employed to examine the cross‐sectional morphology of the joint interface using a JSM‐7500F microscope (JEOL, The Netherlands), operated at an accelerating voltage of 5 kV. Prior to imaging, samples were sputter‐coated with a thin gold layer (approximately 15 nm thickness) using Quorum Q300TD sputter coater (Quorum technologies, UK) to enhance surface conductivity and image resolution.

### Optical Microscopy

A VR‐5000 wide‐area 3D microscope (Keyence, Japan) was used to capture the cross‐section side of the joint.

### Hot Pressing

The hot‐pressed samples in this study were prepared by a heated flat platen press (Joos, press 1000 kN, Gottfried Joos Maschinenfabrik). The samples were taped down between sheets of release film and hot‐pressed between two platens so that only the overlap rea was pressed. The hot‐press process for all the samples was 40 minutes at 180 °C and 3 MPa. For the samples with the only press as manufacturing, large wood veneer sheets were used to spread the ink with a spatula to cover the entire overlap area in a thin layer, and then the samples were cut by a knife.

### Carbon Black Ink

Based on our previous work with the wood flour/CNF/lignin matrix, a plant‐based conductive carbon black (manufactured by Nanografi, 95% purity, 148 nm) was introduced into the base formulation to create conductive inks. The base formulation was maintained at 5 g L^−1^ deionized water, 0.2 g L^−1^ wood flour, 0.1 g L^−1^ CNFs, and 2 g L^−1^ lignin. Four formulations were prepared by varying the carbon black content. For the formulation with 1.25 g CB, the total mass was 8.55 g, corresponding to approximately 58.5 wt% water, 2.3 wt% wood flour, 1.2 wt% CNFs, 23.4 wt% lignin, and 14.6 wt% carbon black. When 0.5 g CB was used, the total mass was 7.8 g (approximately 64.1 wt% water, 2.6 wt% wood flour, 1.3 wt% CNFs, 25.6 wt% lignin, and 6.4 wt% carbon black). The formulation with 1.5 g CB had a total mass of 8.8 g (about 56.8 wt% water, 2.3 wt% wood flour, 1.1 wt% CNFs, 22.7 wt% lignin, and 17.1 wt% carbon black), while the ink with 0.25 g CB totalled 7.55 g (roughly 66.2 wt% water, 2.7 wt% wood flour, 1.3 wt% CNFs, 26.5 wt% lignin, and 3.3 wt% carbon black). All components were homogenized in a planetary mixer (SpeedMixer DAC 150.1 FVZ) at 3500 rpm for 1.5 min using 30s intervals with 15s pauses to prevent overheating, and the dispersions were subsequently degassed by centrifugation (Hermle Z306) at 2000 rpm for 1 min before printing. The ink was 3D printed and air‐dried using the same method mentioned above. Before welding the joint, copper wires were taped to each adherent at distinct positions within the bonding area to ensure electrical contact through the printed ink. After welding, the joint was subjected to mechanical testing using the same setup described earlier, with loading continued until a maximum force of 600 N was reached. The electrical resistance was continuously monitored using a Keithley 2010 multimeter, and the data were subsequently exported for further analysis.

### Continuous Ultrasonic Welding

Continuous ultrasonic welds were produced using a robotic ultrasonic welding system developed by SAMXL (The Netherlands). Welding was performed at an amplitude of 74% (61 µm peak‐to‐peak) and a welding speed of 6 mm s^−1^. A rounded sonotrode (Aeson BV, The Netherlands) with a width of 30 mm and a tip radius of 17.5 mm was employed, providing a gain factor of 3.3. The booster utilized in the system had no additional gain. Welding and consolidation forces were both maintained at 500 N throughout the process.

### Manufacturing of Honeycomb Structure

Honeycomb structures were fabricated from Beech wood veneers using an ultrasonic spot welder (AG RL35, Rinco Ultrasonics, Switzerland). Wood veneers (25 × 125 × 0.4 mm^3^) were initially cut by laser and then reshaped into curved (“hat‐like”) profiles by immersing in warm water and forming around custom‐made 3D‐printed PLA molds, secured with C‐clamps. Subsequently, the formed veneers were dried at 50 °C for 12 h. A three‐cell honeycomb structure was assembled by ultrasonically welding six hat‐shaped veneers at their contact points, applying 80 W ultrasonic power for 3 s per joint.

### Mycelium Growth on Welded Wood Interfaces

Beech wood veneer samples were welded using ultrasonic welding with or without lignin‐based ink. Four groups of samples (*n* = 5 per group) were prepared: 1) welded with lignin ink and inoculated with fungi, 2) welded without ink and inoculated with fungi, 3) welded with lignin ink, no fungi (control), and 4) welded without ink, no fungi (control).

All samples were sterilized with UV light (30 min per side) in a biosafety cabinet. They were briefly dipped in sterile distilled water and placed on a 5 mm layer of agar gel (15 g L^−1^ agar in distilled water) inside sealed plastic boxes. The fungal groups were inoculated with *Ganoderma lucidum* by placing bird seed spawn directly onto the weld interface. The control groups were prepared identically, without inoculation.

The sealed boxes were incubated at 27 °C for 18 d to allow fungal colonization under high humidity. After incubation, samples were removed and immediately imaged and tested in their wet state.

## Conflict of Interest

We are patenting an invention based on these findings.

## Author Contributions

Conceptualization, methodology: M.A., K.W., and K.M.; Software: M.A.; Investigation: M.A., K.W., M.A., N.S.G.‐S, and J.v.S.M.; Validation: All, supervision: K.M.; Writing—original draft: M.A. and K.M.; Writing—review & editing: All

## Supporting information



Supporting Information

Supplemental Video 1

Supplemental Video 2

Supplemental Video 3

Supplemental Video 4

## Data Availability

The data that support the findings of this study are available from the corresponding author upon reasonable request. Source codes will be made available through our GitLab page.

## References

[1] G. Yang , Z. Gong , X. Luo , L. Chen , L. Shuai , Nature 2023, 621, 511. 37553075 10.1038/s41586-023-06507-5 PMC10511307

[2] W. Sun , I. Hafez , B. J. W. Cole , M. Tajvidi , RSC Appl. Interfaces 2024, 1, 1036.

[3] M. I. Maulana , M. A. R. Lubis , F. Febrianto , L. S. Hua , A. H. Iswanto , P. Antov , L. Kristak , E. Mardawati , R. K. Sari , L. H. Zaini , W. Hidayat , V. L. Giudice , L. Todaro , Forests 2022, 13, 1614.

[4] M. Frey , L. Schneider , H. Razi , E. Trachsel , E. Faude , S. M. Koch , K. Masania , P. Fratzl , T. Keplinger , I. Burgert , ACS Sustainable Chem. Eng. 2021, 9, 9638.

[5] H. Zhang , P. Liu , S.Md. Musa , C. Mai , K. Zhang , ACS Sustainable Chem. Eng. 2019, 7, 10452.

[6] P. V. Dhawale , S. K. Vineeth , R. V. Gadhave , J. F. M. J , M. V. Supekar , V. K. Thakur , P. Raghavan , Mater. Adv. 2022, 3, 3365.

[7] I. Vamza , G. Krigers , K. Valters , Environ. Clim. Technol. 2022, 26, 1350.

[8] I. Calvez , R. Garcia , A. Koubaa , V. Landry , A. Cloutier , Curr. For. Rep. 2024, 10, 386. 39301227 10.1007/s40725-024-00227-3 PMC11408402

[9] M. Dunky , in Progress in Adhesion and Adhesives, 1st ed. (Ed: K. L. Mittal ), Wiley, New York 2021, pp. 383–529.

[10] W. Sun , M. Tajvidi , C. Howell , C. G. Hunt , ACS Appl. Mater. Interfaces 2020, 12, 57431. 33306341 10.1021/acsami.0c18165

[11] E. Özdemir , N. Saeidi , A. Javadian , A. Rossi , N. Nolte , S. Ren , A. Dwan , I. Acosta , D. E. Hebel , J. Wurm , P. Eversmann , Biomimetics 2022, 7, 39. 35466256 10.3390/biomimetics7020039 PMC9036262

[12] S. Gantenbein , E. Colucci , J. Käch , E. Trachsel , F. B. Coulter , P. A. Rühs , K. Masania , A. R. Studart , Nat. Mater. 2023, 22, 128. 36550372 10.1038/s41563-022-01429-5

[13] H. Wang , J. Tao , Z. Wu , K. Weiland , Z. Wang , K. Masania , B. Wang , Adv. Sci. 2024, 11, 2309370. 10.1002/advs.202309370 PMC11200020 38477443

[14] I. F. Villegas , J. Thermoplast. Compos. Mater. 2015, 28, 66.

[15] I. F. Villegas , Composites, Part A 2014, 65, 27.

[16] N. S. Guevara‐Sotelo , I. Fernandez Villegas , Materials 2023, 16, 6968. 37959564 10.3390/ma16216968 PMC10650287

[17] A. K. A. Gedara , I. Chianella , J. L. Endrino , Q. Zhang , BioResources 2021, 16, 6448.

[18] A. Pizzi , M. Properzi , J.‐M. Leban , M. Zanetti , F. Pichelin , Maderas, Cienc. Tecnol. 2003, 5, 101.

[19] J. Michel Leban , A. Pizzi , M. Properzi , F. Pichelin , P. Gelhaye , C. Rose , Scand. J. For. Res. 2005, 20, 534.

[20] S. Amirou , A. Pizzi , L. Delmotte , J. Adhes. Sci. Technol. 2020, 34, 13.

[21] S. Stucki , H. Lange , C. H. Dreimol , Y. Weinand , I. Burgert , J. Adhes. Sci. Technol. 2023, 37, 3167.

[22] R. Das , S. S. Joshi , P. S. Owuor , A. Khan , S. Ike , P. Kumar , N. B. Dahotre , C. S. Tiwary , Mater. Today 2025, 87, 125.

[23] L. Salmen , Temperature and Water Induced Softening Behaviour of Wood Fiber Based Materials. (accessed: July 2025)

[24] S. S. Kelley , T. G. Rials , W. G. Glasser , J. Mater. Sci. 1987, 22, 617.

[25] H. Huang , C. Xu , X. Zhu , B. Li , C. Huang , Green Chem. 2023, 25, 4995.

[26] M. Frey , L. Schneider , K. Masania , T. Keplinger , I. Burgert , ACS Appl. Mater. Interfaces 2019, 11, 35305. 31454224 10.1021/acsami.9b11105

[27] F. Meng , Y. Yu , Y. Zhang , W. Yu , J. Gao , Appl. Surf. Sci. 2016, 371, 383.

[28] Ö. Özgenç , S. Durmaz , I. H. Boyaci , H. Eksi‐Kocak , Spectrochim. Acta, Part A 2017, 171, 395. 10.1016/j.saa.2016.08.026 27569772

[29] S. G. Kostryukov , H. B. Matyakubov , Y. Y. Masterova , A. S. Kozlov , M. K. Pryanichnikova , A. A. Pynenkov , N. A. Khluchina , J. Anal. Chem. 2023, 78, 718.

[30] R. Javier‐Astete , J. Jimenez‐Davalos , G. Zolla , PLoS One 2021, 16, 0256559. 10.1371/journal.pone.0256559 PMC8550379 34705842

[31] D. Erçin , Y. Yürüm , J. Anal. Appl. Pyrolysis 2003, 67, 11.

[32] M. Borghei , H. Baniasadi , R. Abidnejad , R. Ajdary , S. Mousavihashemi , D. Robertson , J. Niskanen , E. Kontturi , T. Kallio , O. J. Rojas , Addit. Manuf. 2024, 92, 104397.

[33] M. S. H. Thakur , C. Shi , L. T. Kearney , M. A. S. R. Saadi , M. D. Meyer , A. K. Naskar , P. M. Ajayan , M. M. Rahman , Sci. Adv. 2024, 10, adk3250. 10.1126/sciadv.adk3250 PMC10942110 38489368

[34] Y. Wei , X. Jin , Q. Luo , Q. Li , G. Sun , Composites, Part B 2024, 276, 111225.

[35] P. Hass , F. K. Wittel , P. Niemz , Generic failure mechanisms in adhesive bonds, 2015. (accessed: July 2025)

[36] T. Todorovic , E. Norström , L. Fogelström , E. Malmström , Int. J. Adhes. Adhes. 2024, 135, 103818.

[37] R. Li , L. Li , W. Qiu , D. Y. Zhu , X. Qiu , R. Ou , B. Liu , W. Liu , Adv. Funct. Mater. 2025, 35, 2422605.

[38] Y. Yang , H. Wu , J. Zhang , T. Wen , G. Du , B. Charrier , H. Essawy , A. Pizzi , J. Wu , X. Zhou , X. Chen , Ind. Crops Prod. 2025, 234, 121571.

[39] L. F. Lorenz , C. R. Frihart , J. Appl. Polym. Sci. 2023, 140, 53332.

[40] Y. Xiong , Z. Wu , X. Xi , C. Li , H. Lei , Z. Chen , G. Du , Int. J. Adhes. Adhes. 2025, 138, 103921.

[41] Y. Xiong , Z. Wu , X. Xi , H. Lei , C. Li , Z. Chen , J. Shi , G. Du , Ind. Crops Prod. 2024, 222, 119417.

[42] S. Ghahri , X. Chen , A. Pizzi , R. Hajihassani , A. N. Papadopoulos , Polymers 2021, 13, 595. 33669474 10.3390/polym13040595 PMC7920486

[43] S. Ghahri , B.‐D. Park , Ind. Crops Prod. 2023, 206, 117711.

[44] J. A. Dolan , N. Sathitsuksanoh , K. Rodriguez , B. A. Simmons , C. E. Frazier , S. Renneckar , RSC Adv. 2015, 5, 67267.

[45] R. Das , R. Benjamim , M. Kotal , L. Machado , D. S. Galvao , C. S. Tiwary , Chem. Commun. 2025, 61, 9039. 10.1039/d5cc01464f 40365661

[46] C. H. Dreimol , H. Guo , M. Ritter , T. Keplinger , Y. Ding , R. Günther , E. Poloni , I. Burgert , G. Panzarasa , Nat. Commun. 2022, 13, 3680. 35760793 10.1038/s41467-022-31283-7 PMC9237073

[47] B. Jongbloed , J. Teuwen , G. Palardy , I. Fernandez Villegas , R. Benedictus , J. Compos. Mater. 2020, 54, 2023.

[48] I. H. M. S. Nettersheim , N. S. G. Sotelo , J. C. Verdonk , K. Masania , Compos. Sci. Technol. 2024, 256, 110758.

[49] Q. Sun , Y. Xia , J. Klinger , R. Seifert , J. Kane , V. Thompson , Q. Chen , Powder Technol. 2021, 388, 496.

[50] D14 Committee Test Method for Apparent Shear Strength of Single‐Lap‐Joint Adhesively Bonded Metal Specimens by Tension Loading (Metal‐to‐Metal). (accessed: December 2024)

[51] M. Frey , M. Zirkelbach , C. Dransfeld , E. Faude , E. Trachsel , M. Hannus , I. Burgert , T. Keplinger , JoVE 2019, 153, 60327. 10.3791/60327 31762457

[52] TAPPI , T.A. of the P. and P.I. , TAPPI T 222 om‐02: Acid‐Insoluble Lignin in Wood and Pulp, 2002. (accessed: July 2025)

