# Supplementary material for: Enhancing the Ultrasonic Welding of Wood Using 3D Printed Lignin Energy Directors
Source: Adv Sci (Weinh). 2025 Sep 15;13(15):e07055. doi: 10.1002/advs.202507055 (PMC13042572; doi:10.1002/advs.202507055)
Supplement: Supplementary file 1 — Supporting Information [file ADVS-13-e07055-s005.docx]

# Supplementary Information: Enhancing the ultrasonic welding of wood using three-dimensional printed lignin energy directors

Muhamad Amani^1^, Kathrin Weiland^1^, Mark Ablonczy^1^, Natalia Sofia Guevara-Sotelo^1^, Ioannis Zygouris^1^, Johan van Stuyvesant Meijen^2^, Kunal Masania^1*^

^1^Shaping Matter Lab, Faculty of Aerospace Engineering, Delft University of Technology, Kluyverweg 1, 2629 HS Delft, Netherlands

^2^SAM XL, TU Delft, Rotterdamseweg 382C, 2629 HG Delft, Netherlands

*Corresponding author: [k.masania@tudelft.nl](mailto:k.masania@tudelft.nl)

**Contents:**

Material and methods.

# Figure S1Overview of the experimental design and analytical flow

Figure S2. Overview of ultrasonic welding of wood veneers with the key components

Figure S3. Modulated DSC thermogram of ground beech wood

Figure S4. The temperature build-up on the surface of the joint using a thermal camera

Figure S5. Porosity profile along the sample and across the welded joint

Figure S6. Direct ink writing of lignin ink as energy director and microscopic and SEM images of the cross-section of the joint after welding with ink with and without wood flour

Figure S7. FTIR for inks with and without wood flour compared with without ink

Figure S8. mDSC of lignin inks, lignin and wood flour

Figure S9. TGA data of lignin, lignin inks and beech wood

Figure S10. Failure mode comparison of various wood bonding techniques

Figure S11. Effect of mycelium growth on wet strength of welded wood interface

[**Video S1**](https://surfdrive.surf.nl/files/index.php/s/OL89tp8I0hXn6vD)**.** Ultrasonic wood welding

[**Video S2.**](https://surfdrive.surf.nl/files/index.php/s/CYYxYCvfHPwbBcp) Direct ink writing of the energy director

[**Video S3.**](https://surfdrive.surf.nl/files/index.php/s/T5XDdxYM0yMw0WT) Continuous ultrasonic wood welding

[**Video S4.**](https://surfdrive.surf.nl/files/index.php/s/1biJVnSNQzqgBIE) Honeycomb structure

# Overview of the experimental design and analytical flow

#

Figure S1. The flowchart summarises the stepwise methodology employed in this study, beginning with ultrasonic welding of wood, followed by optimisation of welding parameters and controlling the quality of the joint. These findings were then used to develop an optimised lignin-based ink, which was characterised using rheology, FTIR, mDSC, TGA, and SEM. The performance of the ink was compared with other adhesives under dry and wet conditions. Finally, the potential applications of the ink were demonstrated by adding conductive particles, fabricating complex shapes, and supporting fungal growth.

# Overview of ultrasonic welding of wood veneers with the key components

**
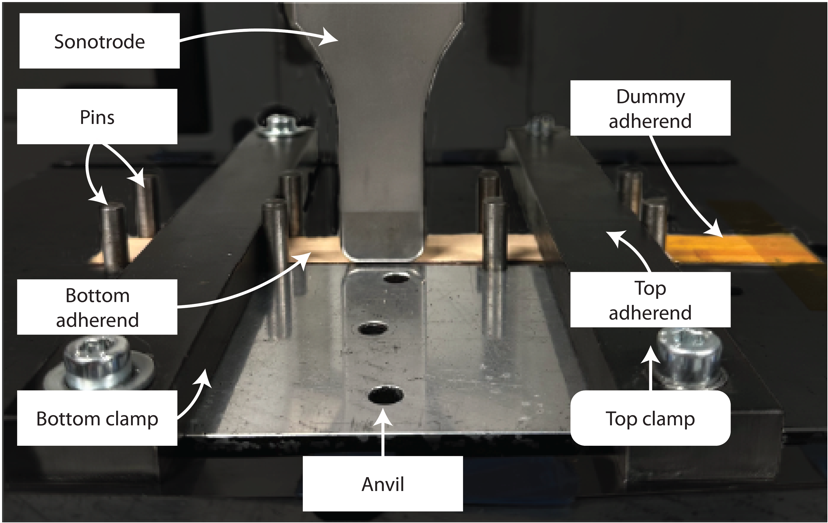
**

Figure S2. Key components and overall view of the ultrasonic welding setup. The sonotrode moves vertically to contact the joint interface, delivering ultrasonic vibrations necessary for welding. The initial position and vertical displacement of the joint are precisely controlled before welding initiation. Pins, top, and bottom clamps ensure stability of both adherends during the welding process, while the dummy adherent prevents deformation of the top adherend, maintain optimal alignment and contact conditions.

# Modulated DSC thermogram of ground beech wood

Figure S3. The plot displays the normalised reversing heat flow (C_p_) versus temperature obtained through modulated differential scanning calorimetry (mDSC) of finely ground beech wood. Exothermic direction is upward. Several key thermal transitions were observed, including moisture evaporation at approximately 101.8 °C and the glass transition (T_g_) at approximately 150 °C, related to the softening of the wood’s amorphous components (primarily lignin and hemicellulose). Further transitions occur at higher temperatures, corresponding to the decomposition of hemicellulose (~250.7 °C), depolymerisation of lignin (~285.3 °C) and degradation of cellulose (~405.8 °C). These thermal signatures reflect the intricate thermal decomposition processes and underlying polymeric interactions in wood, offering insights into its structural and thermomechanical behaviour [24–26].

# The temperature build-up on the surface of the joint using a thermal camera

**
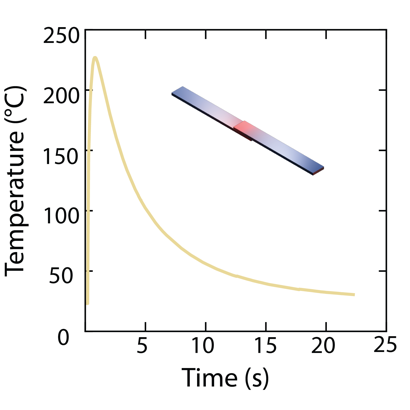
**

Figure S4. Using the thermal camera's keyframes, the joint's temperature build-up was determined using the thermal camera's keyframes. The peak temperature was around 227C at 0.8 seconds.

**Porosity profile along the sample and across the welded joint**

Figure S5. Porosity profile along the sample height obtained from μCT data. A distinct densified region is observed around 15 mm, where porosity decreases notably. Insets show binarised cross-sections at two positions, highlighting the transition from a relatively open porous structure (bottom) to a denser microstructure (top).

# Direct ink writing of lignin ink as energy director and microscopic and SEM images of the cross-section of the joint after welding with ink with and without wood flour


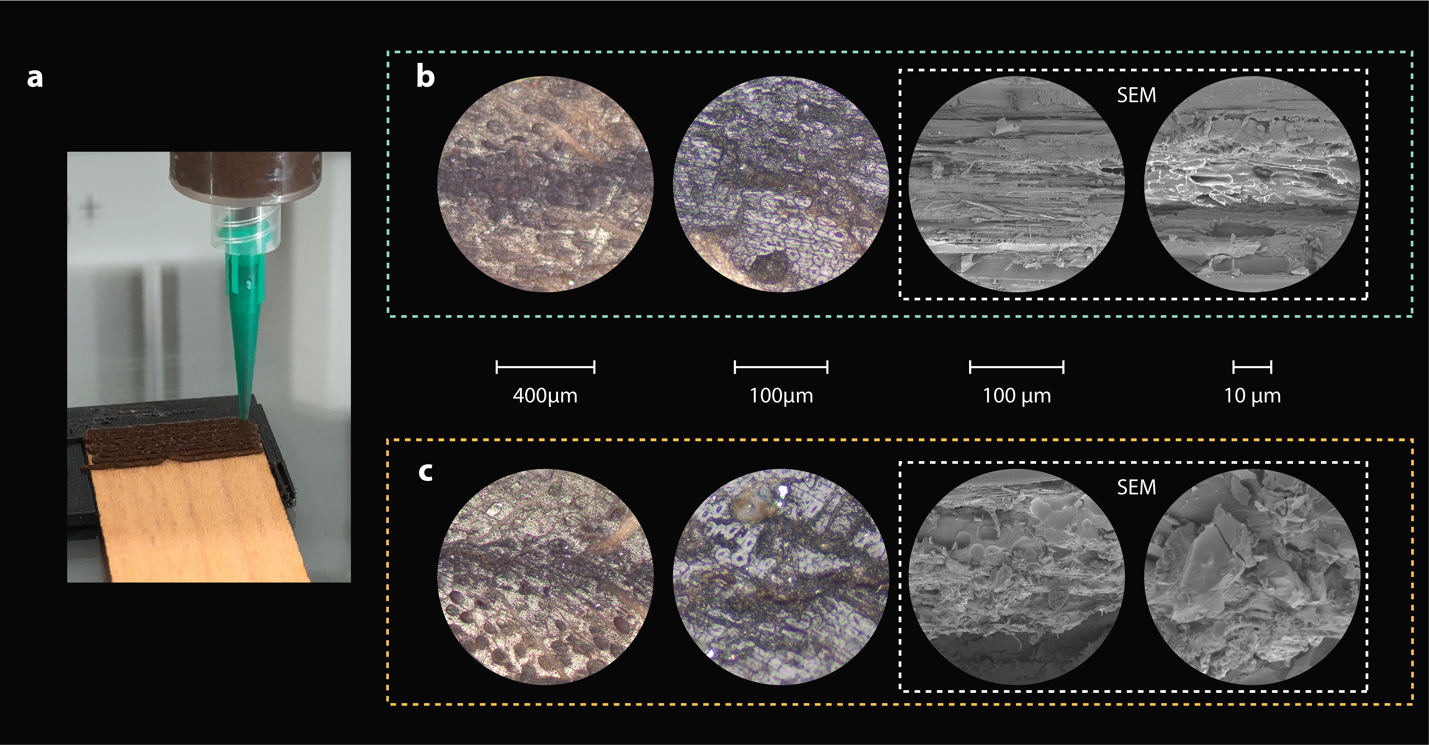


Figure S6. a Direct ink writing of lignin-based ink on the surface of the bottom adherent was performed using a modified 3D printer. b Optical microscopy and SEM images of lignin-based ink without wood flour. The cross-section of the joint shows clear evidence of cellular compression and densification at the interface. The increased local density of lignin indicated effective fusion between the lignin-based ink and the native lignin within the wood cell walls, confirming successful interactions under pressure and elevated temperature. c Optical microscopy and SEM images of lignin-based inks with wood flour revealed a sparse distribution of wood flour particles due to their comparatively low content relative to other ink components. Nevertheless, the existing wood flour particles displayed preferential alignment along the compression axis at the interface, reflecting directional deformation and particle orientation resulting from the applied mechanical pressure during welding.

# FTIR for inks with and without wood flour compared with and without ink

**
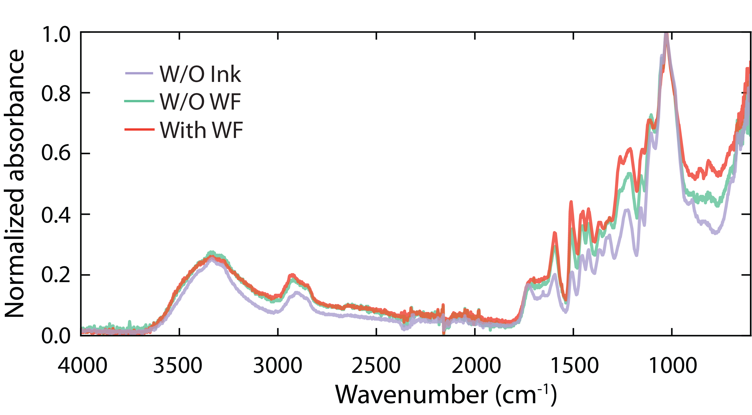
**

Figure S7. Spectra from joints without ink, with lignin-based ink without wood flour, and lignin-based ink containing wood flour are compared. The spectra clearly show increased absorption intensities for joints incorporating lignin-based inks, indicating greater organic content at the bonded interface. However, no distinct spectral differences emerge when wood flour is introduced into the ink, suggesting that wood flour addition does not significantly alter the chemical characteristics detectable by FTIR analysis.

# mDSC of lignin inks, lignin and wood flour

Figure S8. mDSC thermograms of lignin-based inks compared with kraft lignin and beech wood flour. Exothermic direction is plotted upward. Normalised heat flow profiles demonstrate the influence of wood flour incorporation on the thermal behaviour of lignin inks. The presence of wood flour shifts the thermal transitions toward lower temperatures, indicating improved compatibility within the composite matrix. Kraft lignin exhibits a characteristic broad endothermic valley in this region, attributed to its heterogeneous and amorphous structure. The addition of wood flour induces a slight downward shift in T_g_ in the ink formulations, suggesting enhanced molecular interaction or plasticization.

# TGA data of lignin, lignin inks and beech wood

**
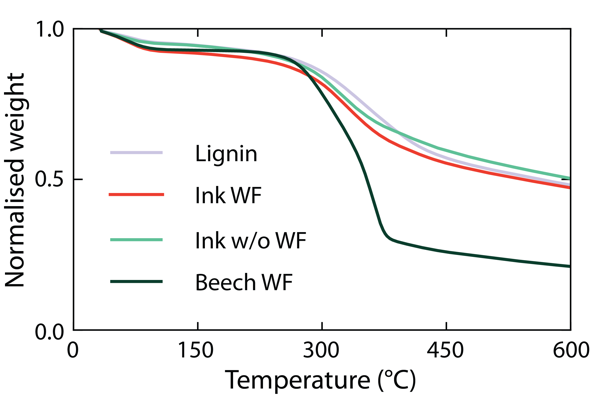
**

Figure S9. Normalised weight loss profiles illustrate distinct thermal decomposition behaviours. Wood flour exhibits significant mass loss at lower temperatures. Whereas adding lignin enhances thermal stability in inks, shifting the degradation onset to higher temperatures.

**Failure mode comparison of various wood bonding techniques**

Figure S10. Fracture surfaces of lap-shear specimens bonded using ultrasonic welding with lignin-based ink with wood flour, wood glue, epoxy, and cyanoacrylate, showing distinct failure modes. Green indicates stock-break failure in the wood substrate, red denotes cohesive failure within the adhesive, and blue highlights adhesive failure at the bond line. Ultrasonic welding and epoxy show stock-break failure predominantly at edge regions (bathtub effect), while wood glue demonstrates cohesive failure. Cyanoacrylate shows both adhesive and stock-break failure due to rapid setting and reduced interfacial wetting.

**Effect of mycelium growth on wet strength of welded wood interface**

Figure S11. Wet shear strength of welded beech veneer samples after 18 days of incubation under high-humidity, mycelium-growing conditions. Only samples welded with lignin-based ink remained mechanically stable, while those without ink disintegrated and could not be tested. No significant difference in strength was observed between fungus-inoculated and uninoculated ink groups, indicating that the welded interface remains robust even under fungal colonization. Group labels: I+F (USW lignin ink with mycelium growth), NI+F (USW without ink and with mycelium growth), I–F (USW lignin ink with mycelium growth), NI–F (USW without lignin ink and without mycelium growth).
